# A core component of psychological therapy causes adaptive changes in computational learning mechanisms

**DOI:** 10.1017/S0033291723001587

**Published:** 2024-01

**Authors:** Quentin Dercon, Sara Z. Mehrhof, Timothy R. Sandhu, Caitlin Hitchcock, Rebecca P. Lawson, Diego A. Pizzagalli, Tim Dalgleish, Camilla L. Nord

**Affiliations:** 1 MRC Cognition and Brain Sciences Unit, University of Cambridge, Cambridge, UK; 2 UCL Institute of Mental Health, University College London, London, UK; 3 Department of Psychology, University of Cambridge, Cambridge, UK; 4 Melbourne School of Psychological Sciences, University of Melbourne, Melbourne, Australia; 5 Department of Psychiatry, Harvard Medical School, Boston, MA, USA; 6 Center for Depression, Anxiety, and Stress Research, McLean Hospital, Belmont, MA, USA; 7 Cambridgeshire and Peterborough NHS Foundation Trust, Cambridgeshire, UK

**Keywords:** Computational psychiatry, distancing, emotion regulation, psychotherapy, reinforcement learning

## Abstract

**Background:**

Cognitive distancing is an emotion regulation strategy commonly used in psychological treatment of various mental health disorders, but its therapeutic mechanisms are unknown.

**Methods:**

935 participants completed an online reinforcement learning task involving choices between pairs of symbols with differing reward contingencies. Half (49.1%) of the sample was randomised to a cognitive self-distancing intervention and were trained to regulate or ‘take a step back’ from their emotional response to feedback throughout. Established computational (*Q*-learning) models were then fit to individuals' choices to derive reinforcement learning parameters capturing clarity of choice values (inverse temperature) and their sensitivity to positive and negative feedback (learning rates).

**Results:**

Cognitive distancing improved task performance, including when participants were later tested on novel combinations of symbols without feedback. Group differences in computational model-derived parameters revealed that cognitive distancing resulted in clearer representations of option values (estimated 0.17 higher inverse temperatures). Simultaneously, distancing caused increased sensitivity to negative feedback (estimated 19% higher loss learning rates). Exploratory analyses suggested this resulted from an evolving shift in strategy by distanced participants: initially, choices were more determined by expected value differences between symbols, but as the task progressed, they became more sensitive to negative feedback, with evidence for a difference strongest by the end of training.

**Conclusions:**

Adaptive effects on the computations that underlie learning from reward and loss may explain the therapeutic benefits of cognitive distancing. Over time and with practice, cognitive distancing may improve symptoms of mental health disorders by promoting more effective engagement with negative information.

## Introduction

Emotion regulation difficulties occur across psychiatric disorders, improve following effective psychological treatment (Sloan et al., 2017), and may predict treatment response (Siegle, Carter, & Thase, 2006). Cognitive distancing is a core therapeutic strategy to facilitate emotion regulation, forming a central component of cognitive behavioural therapy (CBT) (Papa, Boland, & Sewell, 2012), mindfulness-based cognitive therapy (Hamidian, Omidi, Mousavinasab, & Naziri, 2016), and dialectical behaviour therapy (Neacsiu, Eberle, Kramer, Wiesmann, & Linehan, 2014), among others. When cognitive distancing, patients are encouraged to view negative thoughts from afar, such as by ‘adopt[ing] the position of a neutral observer’ (Staudinger, Erk, Abler, & Walter, 2009). With practice, distancing promotes disengagement from intense emotions in favour of a more experiential perspective, reducing distress (Mennin, Ellard, Fresco, & Gross, 2013) and depressed thoughts (Kross, Gard, Deldin, Clifton, & Ayduk, 2012).

Despite major advances in understanding the computations underpinning pharmacological treatments in psychiatry, whether these mechanisms parallel those involved in psychological treatment is unknown. Reward learning is compromised in numerous psychiatric disorders (Halahakoon et al., 2020; Huys, Pizzagalli, Bogdan, & Dayan, 2013; Maia & Frank, 2011), and is a target of pharmacological interventions for conditions including depression (Hales, Houghton, & Robinson, 2017), schizophrenia (Insel et al., 2014), and bipolar disorder (Volman et al., 2020). Reward learning may also be a target of psychological interventions (Delgado, Gillis, & Phelps, 2008). CBT has been shown to directly affect reward learning (Brown et al., 2021), altering how patients assign value to actions and the extent to which these are updated by different environmental signals. These computations are formalised in a reinforcement learning framework as expected values (EVs) and prediction errors (PEs) respectively (Sutton & Barto, 1998).

Cognitive distancing has been specifically shown to affect midbrain reward representations through top-down prefrontal cortex input (Staudinger et al., 2009; Staudinger, Erk, & Walter, 2011). However, the computational underpinnings of these representations (Huys, Maia, & Frank, 2016) are not known. For example, does cognitive distancing alter the extent to which PEs update the EV of future decisions (learning rate), or does distancing alter the extent to which these EVs drive decisions (inverse temperature)? Here, in a large online sample (*n* = 995) broadly representative of the UK population, we test whether and how cognitive distancing alters reinforcement learning, using a probabilistic selection task (PST) (Frank, Seeberger, & O'Reilly, 2004; Frank, Moustafa, Haughey, Curran, & Hutchison, 2007) combined with established computational models to decompose components of participant behaviour. Our study methods and analyses were preregistered (https://osf.io/fd4qu).

## Methods and materials

### Recruitment and study design

**Figure 1.**
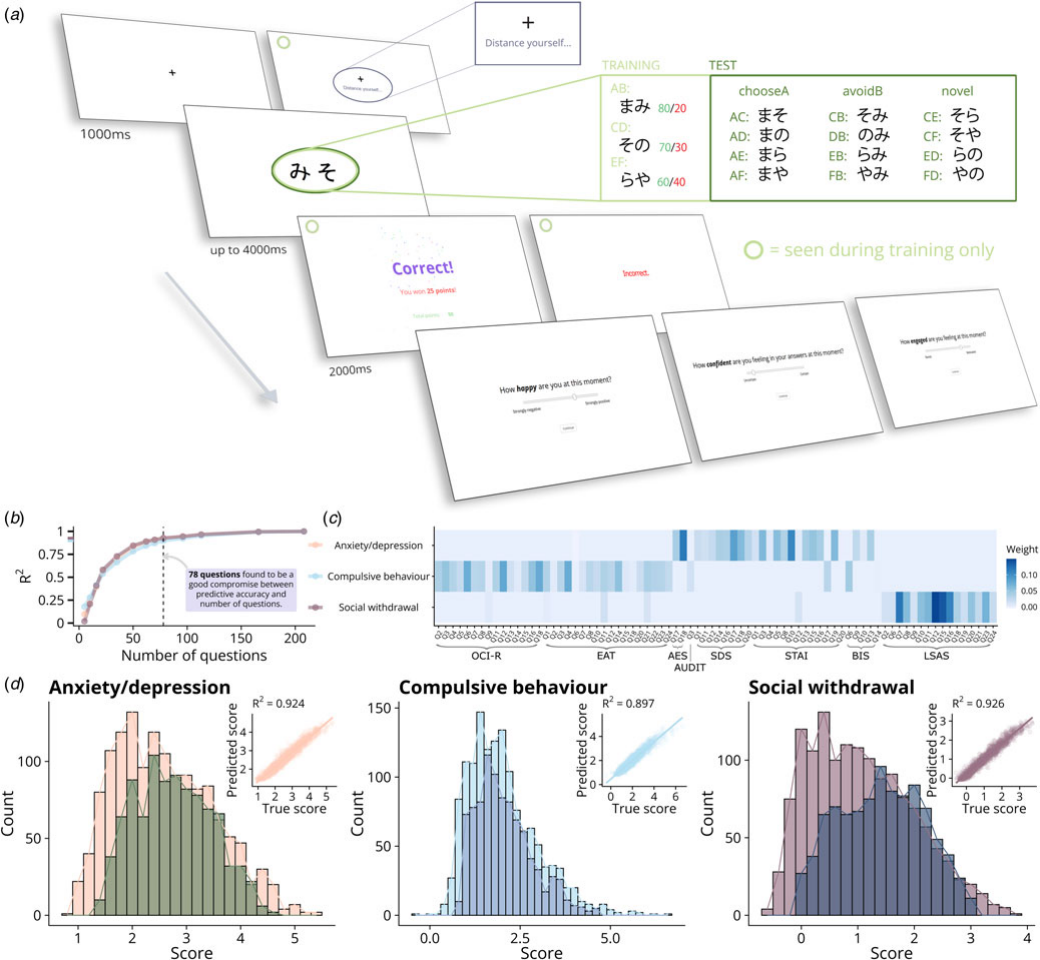
Reinforcement learning task design, transdiagnostic factor derivation, and comparison of factor scores to previous samples. a. The probabilistic selection task (PST) in the present study consisted of six blocks of sixty trials (training phase) where participants were instructed to choose between Hiragana characters presented as three pairs (AB, CD, EF; twenty of each per block), and given feedback (‘Correct!’ or ‘Incorrect.’). One character in each of the pairs was consistently more likely to be correct (reward probabilities of 0.8/0.2, 0.7/0.3, and 0.6/0.4 for A/B, C/D, and E/F respectively. 497 participants (49.9%) were randomised to a self-distancing intervention, and additionally received a prompt to ‘Distance yourself…’ with the fixation cross at the start of each training trial. The training phase was followed by a sixty-trial test phase without feedback where the twelve other possible character combinations were added (i.e. four of each of the fifteen pairs). All participants saw the same six characters, but the pairs themselves were randomised for each participant, and the order of the pairs was counterbalanced across trials. One of three affect questions was asked after each trial, with unlimited time to answer. b. Multi-target lasso regression with five-fold cross-validation was used to predict the three transdiagnostic dimension factor scores from different subsets of the 209 questions in the original dataset (Gillan et al., 2016, study 2). 78 questions were found to predict the three factor scores with high predictive accuracy (colours correspond to heatmap labels). c. Heatmap of five-fold cross-validated multi-target lasso regression coefficient weights for each of the included 78 questions across eight psychiatric questionnaires used to predict the three transdiagnostic symptom dimensions (i.e. weights for all other questions fixed at zero; see online Supplementary Methods for full details). d. Comparison between the factor score distributions for the *n* = 935 non-excluded participants in the present study (darker colours), and those previously obtained by Gillan et al. (2016) in *n* = 1413 participants (lighter colours). Inset plots show the predictive validity of the subset of 78 questions in predicting these scores in the original dataset.

Participants were recruited on Prolific (Palan & Schitter, 2018) over three weeks in April–May 2021. Prolific pre-screeners were used to recruit batches of male and female UK nationals with and without a self-reported prior diagnosis of a psychiatric disorder across five age-groups (18–24, 25–34, 35–44, 45–54, and 55+), with target numbers for each of the twenty batches calculated based on UK population data (Office of National Statistics, 2016; Stansfeld et al., 2016) (see online Supplementary Methods). The online study was written in JavaScript using the jsPsych library (de Leeuw, 2015), and consisted of a reinforcement learning task (Fig. 1a), followed by a working memory task (digit span; see online Supplementary Methods) and a psychiatric questionnaire battery. A total of 995 participants completed all study components. Participants were paid a fixed rate of £9 (approximately £6/h), plus a bonus contingent on task performance to encourage engagement (£1 if in the top 30% of points or £2 in the top 10%). The study was approved by the University of Cambridge Human Biology Research Ethics Committee (HBREC.2020.40) and jointly sponsored by the University of Cambridge and Cambridge University Hospitals NHS Foundation Trust (IRAS ID 289980). All participants provided written informed consent through an online form, in line with approved University of Cambridge Human Biology Research Ethics Committee procedures for online studies.

#### Exclusion criteria

Exclusion criteria included self-reported diagnosis of a neurological disorder (*n* = 18); English proficiency below B2 level (good command or working knowledge; *n* = 3); and a digit span of 0 (*n* *=* 5). Based on recent recommendations (Zorowitz, Solis, Niv, & Bennett, 2021), two harder catch questions were included in the psychiatric questionnaire battery (e.g. ‘In the past week, I would (have) avoided… Eating mouldy food’; expected answer ‘Usually’), in addition to two standard catch questions (e.g. ‘Please answer ‘Strongly Agree’ to this question.’). Participants were excluded if they got one of the standard questions wrong (*n* = 16), or both harder questions wrong (*n* = 20).

### Reinforcement learning task

In the present study, the PST (Frank et al., 2004, 2007) consisted of a training (six blocks of sixty trials) and test phase (sixty trials; Fig. 1a). Prior to starting the task, all participants viewed a one-minute instructional video, and had to correctly answer a multiple-choice question on these instructions. Participants randomised to the control arm (*n* = 498) then proceeded to the first training trial, while participants randomised to the intervention (*n* = 497) were also shown a second ninety-second video (see ‘Cognitive distancing manipulation' below) before advancing. On each trial, participants were presented with one of three pairs of Japanese Hiragana characters (there were exactly twenty of each pair per block) and instructed to choose the left or right character via key press. The symbols within each stimulus pair were associated with different chances of reward: symbol ‘A’ was correct 80% of the time, and ‘B’ 20% of the time, with respective probabilities of 0.7/0.3 and 0.6/0.4 for the ‘C’/‘D’ and ‘E’/‘F’ pairs. Participants were instructed to pick the symbol they felt was most likely correct. After receiving feedback (‘Correct!’ or ‘Incorrect.’, plus twenty-five points if the answer was correct), participants were asked to indicate their current feelings (0–100) with respect to one of three affect adjectives (happy/confident/engaged), with unlimited time to answer. The training phase was followed by a sixty-trial test phase without feedback, which included the twelve other possible character combinations. All participants saw the same six characters, but the pairs themselves were randomised for each participant, and the order of the pairs was counterbalanced across trials. Twelve participants (1.2%) reported prior familiarity with Hiragana characters.

### Cognitive distancing manipulation

Half of the sample (*n* = 497) was randomly allocated to the cognitive distancing manipulation. After viewing the instructional video and correctly answering the multiple-choice question on these instructions as detailed above, participants randomised to the cognitive distancing intervention were also shown a second ninety-second video. In this video, the concept of cognitive distancing was introduced as ‘the ability to take mental ‘step back’ from your immediate reactions to events, and view these events from a broader, calmer, and less emotional perspective’, and participants were suggested to practice it by trying to ‘imagine yourself as an external observer, watching yourself perform the task from a distance.’ It was then explained that they should still try to win as many points as possible, but that whenever they had an emotional response to a trial, they should ‘try to distance yourself from your immediate reaction, by taking a step back from how you are feeling.’ Following these instructions (see online Supplementary Methods for full text), the task proceeded in the same manner to that taken by non-distanced participants, except that they received an additional reminder to ‘Distance yourself…’ with the fixation cross that preceded each training trial (Fig. 1a). In the test phase, this reminder was omitted, as there was no feedback.

### Psychiatric questionnaires and transdiagnostic factor derivation

After completing the reinforcement learning task, all participants completed a psychiatric questionnaire battery. Using an approach recently termed computational factor modelling (Wise, Robinson, & Gillan, 2022), we estimated scores for participants on each of three well-characterised transdiagnostic symptom dimensions – anxiety/depression, compulsive behaviour, and social withdrawal (Gillan, Kosinski, Whelan, Phelps, & Daw, 2016) – which could then be related to learning parameters. Though these scores were originally derived from 209 individual questionnaire items, we used a method described previously (Wise & Dolan, 2020) to identify a subset of items which could predict scores in the original dataset (Gillan et al., 2016) with high accuracy. Specifically, a multi-target lasso regression model was trained on the raw question ratings of Gillan et al.'s original dataset (study 2) with the three factor scores as the responses, with differing numbers of question coefficients fixed to zero. Five-fold cross-validation was then used to assess the predictive accuracy of these question subsets (Wise & Dolan, 2020). We found that 78 questions from eight different questionnaires (online Supplementary Table S1), represented a good compromise between number of questions and predictive accuracy (*R*^2^ ≥ 0.9 for all three dimensions; Fig. 1b). Coefficient estimates from the multi-target lasso regression model fit to the original dataset (Fig. 1c) were then used to predict factor scores on each of the transdiagnostic symptom dimensions for all individuals in our sample, based on their answers to the included 78 questions (Fig. 1d).

### Computational modelling

#### Q-learning models

Model-free reinforcement learning in the PST is commonly modelled using *Q*-learning models (Watkins & Dayan, 1992) with single or dual learning rates (*α*) (Frank et al., 2007). The key distinction is that, in the dual learning rate model, EVs (termed *Q*-values) are assumed to update at different rates depending on whether the feedback is negative (i.e. reward *r*_*t*_ = 0, so PE *δ*_*t*_ < 0; termed *α*_*loss*_) or positive (i.e. reward *r*_*t*_ = 1, so PE *δ*_*t*_ ≥ 0; termed *α*_*reward*_). Both models assume that EVs assigned to the stimuli in each pair are updated in response to feedback; higher learning rate parameter values (*α*) indicate increased sensitivity of choices to recent feedback (single learning rate), or in dual learning rate models, differential sensitivity to positive or negative feedback (Frank et al., 2007) (reward or loss learning rate; *α*_*reward*_ or *α*_*loss*_). These EVs are converted to probabilities of choosing one symbol over another via a softmax logistic function, with the extent to which differences in *Q*-values determined choices weighted by an inverse temperature parameter (*β*) (higher values suggest choices more determined by EV differences). In addition to modelling ‘training alone’, these models can also be extended to include test trials (‘training-plus-test’). In the absence of feedback, EVs are assumed to be fixed at the end of training, but in these models, the log probability density was additionally incremented on test phase choices. As such, parameter values from these models are interpreted as those which best reflect choices across training plus those made in the subsequent test phase. See online Supplementary Methods for further details.

#### Model fitting and outcome generalised linear models

Models were fit in a hierarchical Bayesian manner (Ahn, Krawitz, Kim, Busemeyer, & Brown, 2011; Gelman et al., 2013) via CmdStan (Stan Development Team, 2021), separately for distanced and non-distanced participants (Valton, Wise, & Robinson, 2020). Stan model code was adapted from the hBayesDM (Ahn, Haines, & Zhang, 2017) repository. Models were fit using Markov chain Monte Carlo (MCMC) with four parallel chains, and 4000 warm-up plus 20 000 sampling draws per chain. Numerical and visual MCMC diagnostics, including expected sample size (ESS) and split R-hat (Vehtari, Gelman, Simpson, Carpenter, & Burkner, 2021) were used to assess chain mixing and convergence. Individuals' parameters were omitted from subsequent analyses if they had either split R-hat ⩾ 1.1 or bulk ESS < 100 for any parameter (this applied to no more than two individuals per fit). Model posterior predictions were also compared to observed choices for all individuals, and the ability of all models to recover known parameter values from simulated data was also assessed (see online Supplementary Methods). Models were compared using two numerical metrics of out-of-sample predictive accuracy: expected log posterior density (ELPD; higher is better), and the leave-one-out information criterion (LOOIC; lower is better) (Vehtari, Gelman, & Gabry, 2016).

**Figure 2.**
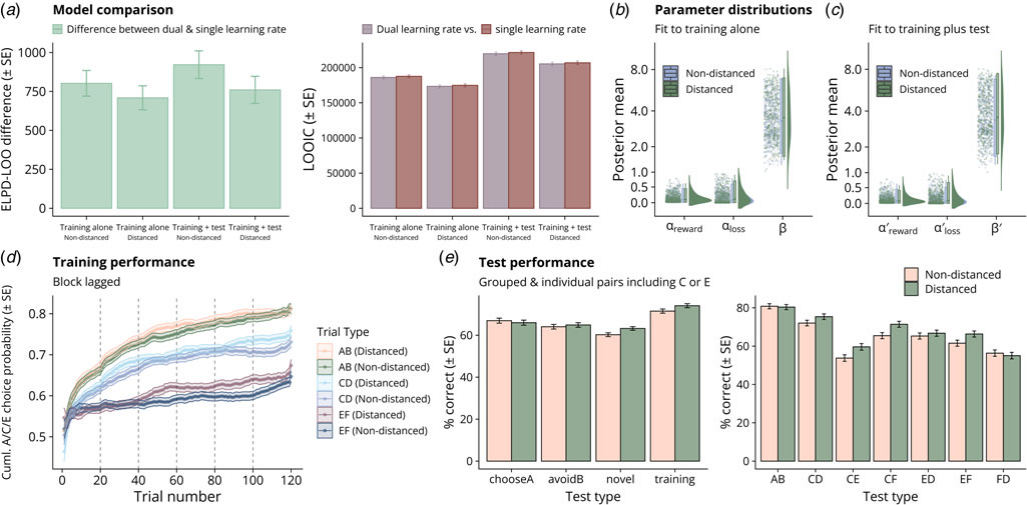
Model comparison, parameter distributions, and raw training and test phase performance. a. Difference in numerical fit metrics between the dual and single learning rate models fit to training data alone, or training-plus-test, by distancing group. ELPD is the expected log posterior density (presented here as the difference between the dual and single learning rate models, where positive differences indicate a better model), and LOOIC is the leave-one-out information criterion (lower indicates a better model). b–c. Distributions of individual-level posterior means for learning parameters from the fits to training (b) and test (c) data. d. Raw training performance (cumulative probability of choosing the higher-probability symbol A/C/E), by group and stimulus pair, lagged by twenty trials (i.e. block-lagged, as each pair is presented twenty times per block. e. Raw test phase performance (% correct, where ‘correct’ is choosing the option in each pair which was most often correct during training) by test type, plus test phase performance on individual training pairs and novel pairs including symbols C or E.

To quantify the evidence for associations between model parameters and cognitive distancing or transdiagnostic factor scores, Bayesian GLMs were fit via CmdStan (Stan Development Team, 2021), using Stan models and priors from the rstanarm R package (Goodrich, Gabry, Ali, & Brilleman, 2020). Due to positively skewed distributions (Fig. 2b, c), the association between learning rate(s) and cognitive distancing was assessed using gamma family GLMs with log link functions, while the association between the inverse temperatures and outcomes was assessed using standard linear regression. All models were adjusted for age, gender (male or female; imputed as natal sex for non-binary individuals given low numbers), and digit span, with models relating parameters to transdiagnostic factor scores additionally adjusted for distancing status.

An exploratory analysis was then run to assess at what stage differences in training performance emerged between distanced and non-distanced participants. To do so, the dual learning rate *Q*-learning model was fit separately in each group as before to trials from increasing numbers of training blocks (i.e. block one, block one to two, block one to three, etc.). The inclusion of all previous trials in each model is necessary as the beginning of training is the only time we can assume *Q*-values for all symbols to be zero. Subsequently, Bayesian GLMs were run to assess the evidence for group differences in learning parameters at each stage as before.

## Results

After applying exclusion criteria, a total of 935 participants (49.1% distanced) were included in analyses (Table 1). Dual learning rate models had consistently higher ELPD and lower LOOIC than corresponding single learning rate models (Fig. 2a), indicating better estimated out-of-sample predictive accuracy, though we report results from all models, as they are largely comparable. Across all non-excluded participants, mean compulsive behaviour factor scores estimated from psychiatric questionnaires were comparable to a similarly-large online sample (2.05 *v.* 2.00; *t*(2286.1) = 1.24, *p* = 0.22) (Gillan et al., 2016); mean scores for the anxiety/depression factor were slightly higher (2.76 *v.* 2.59; *t*(2287) = 5.04, *p* < 0.001); and mean scores for social withdrawal markedly higher (1.41 *v.* 1.06; *t*(2225.7) = 10.2, *p* < 0.001) (Fig. 1d).

**Table 1.** Demographic characteristics of the sample, by group.

	Non-distanced	Distanced	Excluded
Cohort size	476	459	60
**Demographics**			
Age, mean (standard deviation (s.d.); range)	45.3 (14.9; 18–86)	45.5 (15.6; 18–83)	40.0 (16.1; 18–74)
Gender, number (%)			
Male	231 (48.5)	229 (49.9)	25 (41.7)
Female	243 (51.1)	229 (49.9)	33 (55.0)
Non-binary	2 (0.4)	1 (0.2)	2 (3.3)
Ethnicity, number (%)			
White	431 (90.5)	405 (88.2)	51 (85.0)
Asian	23 (4.8)	28 (6.1)	6 (10.0)
Black	9 (1.9)	9 (2.0)	2 (3.3)
Mixed	12 (2.5)	13 (2.8)	1 (1.7)
Other	1 (0.2)	4 (0.9)	0 (0.0)
Subjective socioeconomic status (/9), median (interquartile range (IQR))	5 (4–6)	5 (4–6)	5 (4–6)
Intermediate or lower (⩽B1) English proficiency, number (%)	0 (0.0)	0 (0.0)	3 (3.3)
Can read Hiragana characters, number (%)	3 (0.6)	9 (2.0)	0 (0.0)
**Comorbidities**			
Neurological disorder, number (%)	0 (0.0)	0 (0.0)	18 (30.0)
Psychiatric disorder (ever diagnosed), number (%)			
Generalised or social anxiety disorder	97 (20.4)	101 (22.0)	16 (26.7)
Major depressive disorder	33 (6.9)	37 (8.1)	5 (8.3)
Substance dependence	2 (0.4)	6 (1.3)	0 (0.0)
Any	122 (25.6)	132 (28.8)	20 (33.3)
Current medications, number (%)			
Antidepressant (any)	61 (12.8)	69 (15.0)	14 (23.3)
Anti-hypertensive, anti-coagulant, or statin	49 (10.3)	62 (13.5)	6 (10.0)
Contraceptive pill	24 (5.0)	26 (5.7)	2 (3.3)
Painkiller	25 (5.3)	30 (6.5)	11 (18.3)
**Task performance**			
Time taken (minutes), mean (s.d.)	93.9 (25.1)	93.9 (25.5)	90.6 (24.0)
Mean RT during training, mean (s.d.)	1117.8 (356.1)	1157.9 (363.7)	1002.0 (344.4)
Digit span, median (IQR)	7 (6–8)	7 (6–8)	6.5 (6–8)

### Group differences in raw training and test phase performance, and affect ratings

We assessed whether cognitive distancing affected learning during the task. When comparing the performance of the groups over the course of training, quantified by the mean proportion of times participants chose the symbol most likely to be correct in each pair in each training block, we found strong evidence that the groups differed in accuracy across training trials (multivariate block-stratified Kruskal–Wallis 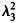 = 18.6, *p* = 0.0003), with distanced individuals appearing to perform better (Fig. 2d). Though post-hoc testing indicated some weak evidence that distanced individuals performed on average 1.40% better on the easiest ‘AB’ pairs across training blocks (reward probabilities 0.8/0.2; block-stratified Kruskal–Wallis 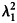 = 4.17, Holm–Bonferroni corrected *p* = 0.041), the evidence was stronger for more ‘difficult’ pairs, where the symbols' reward probabilities were closer together (Fig. 2d, e). Specifically, distanced participants performed on average 2.65% better on medium-difficulty ‘CD’ trials (reward probabilities 0.7/0.3; block-stratified Kruskal–Wallis 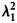 = 11.1, Holm–Bonferroni corrected *p* = 0.003), and on average 3.09% better on the most difficult ‘EF’ pairs in each block (reward probabilities 0.6/0.4; block-stratified Kruskal–Wallis 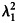 = 11.2, Holm–Bonferroni corrected *p* = 0.003).

In the test phase, there was only trend-level evidence that the groups differed in performance over the four groups of stimuli (multivariate Kruskal–Wallis 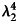 = 7.73, *p* = 0.10). Post-hoc testing suggested that while there was little evidence of any group difference in performance on test pairs including the most-likely correct ‘A’ symbol or the least-likely ‘B’ symbol (‘chooseA’ or ‘avoidB’ respectively), there was some weak evidence that distanced individuals also performed on average 5.0% better when tested on difficult novel pairs (those excluding the symbols most or least associated with reward), though the evidence for this was trend-level after multiple comparisons adjustment (Kruskal–Wallis 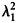 = 4.87, Holm–Bonferroni corrected *p* = 0.11; Fig. 2e).

Distancing also had subtle effects on self-reported affect throughout training. Linear mixed effects models were used to relate average affect ratings per block to group, block, and their interaction (adjusting for age, gender, and digit span). All affect ratings decreased over time, but there was evidence for a block-by-distancing interaction for happiness and engagement (but not confidence), such that both happiness and engagement declined by an estimated 0.44 and 0.41 points (out of 100) less per block in distanced participants (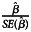 = 2.95, 95% CI (0.14–0.73); and 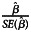 = 2.22, 95% CI (0.05–0.78) for happiness and engagement respectively).

### Associations between learning parameters and transdiagnostic symptom dimensions

Individual-level posterior mean parameter values from single or dual learning rate *Q*-learning models fit to training alone or training-plus-test (parameters from fits to training-plus-test denoted prime) were related to the three transdiagnostic factors using Bayesian GLMs adjusted for age, gender, digit span and distancing status.

**Figure 3.**
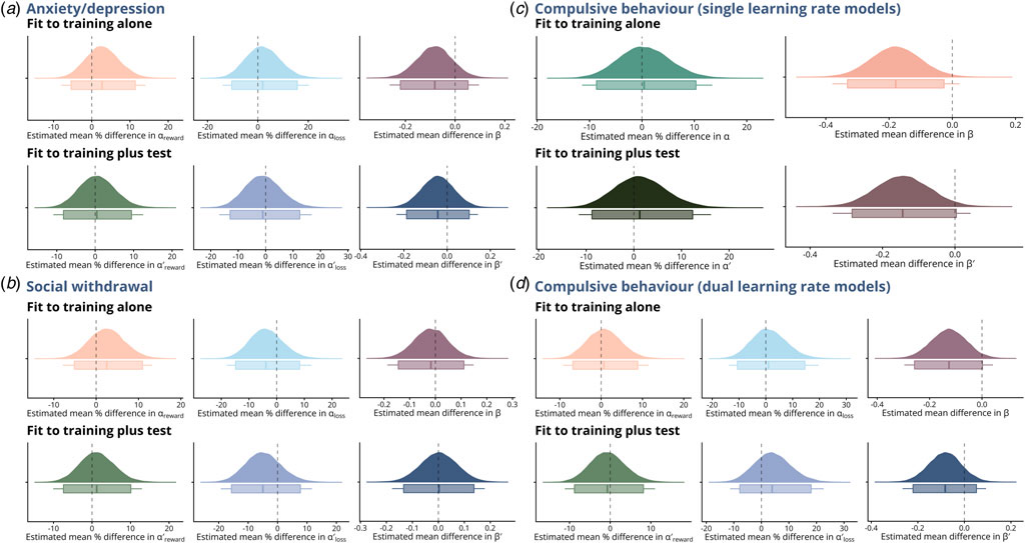
Associations between reinforcement learning parameters and transdiagnostic psychiatric symptom dimensions. a–d. Coefficient posterior distributions from Bayesian GLMs (adjusted for age, gender, digit span and distancing status) reflecting the estimated percentage change in the learning rate parameter or the estimated mean change in the inverse temperature for a unit increase in anxiety/depression (a), social withdrawal (b), and compulsive behaviour (c–d) transdiagnostic factor scores. Parameters were derived from single (only compulsive behaviour presented here, C) or dual learning rate (a–b & d) *Q*-learning models fit to training alone or training-plus-test (parameters from fits to training-plus-test denoted prime). In all plots, boxplot boxes denote 95% HDI, and lines denote 99% HDI.

Results from the GLMs suggested there was limited evidence for an association between any *Q*-learning model parameter and scores on either the anxiety/depression or social withdrawal factors (Fig. 3a, b). An exception was the negative association between the anxiety/depression factor score and the inverse temperature parameter *β* (from the dual learning rate model fit to training alone), such that unit increases in this score were associated with lower *β*, indicating lower sensitivity to value differences between symbols in those scoring higher on this transdiagnostic symptom dimension (Fig. 3a; posterior mean coefficient = −0.08). Still, the evidence for this was very weak, with the 95% highest density interval (HDI) wide and including zero (−0.22, 0.05).

There was also little evidence of any association between *β*^′^ and anxiety/depression from models fit to training-plus-test, nor of associations between learning rate parameters from any model and any of the three factor scores. That said, we did find evidence that increases in compulsive behaviour factor scores were associated with lower inverse temperature values (*β* and *β*^′^; Fig. 3c, d). The strongest evidence for this came from the single learning rate models (Fig. 3c) [*β*: 95% HDI = (−0.33, −0.03); *β*^′^: 95% HDI = (−0.28, 0.004)]; evidence from the dual learning rate model was consistent but weaker (Fig. 3d) [*β*: 95% HDI = (−0.26, 0.002); *β*^′^: 95% HDI = (−0.22, 0.05)].

### Associations between learning parameters and cognitive distancing

To quantify potential differences in *Q*-learning model parameters between groups, we compared individual-level posterior means between distanced and non-distanced individuals using adjusted Bayesian GLMs.

There was weak but consistent evidence that distanced participants had higher inverse temperature values (*β*), suggesting less stochastic choices throughout training, with the mean difference estimated at 0.17 from both models [95% HDI = (−0.05, 0.39) and (−0.01, 0.37) for single and dual learning rate models, respectively]. Distanced participants also showed increased sensitivity to recent feedback, as indicated by estimated 15.3% higher *α* values [single learning rate model; 95% HDI for multiplier = (1.01, 1.32); Fig. 3a]. Notably, the dual learning rate model showed this increased sensitivity was specific to negative feedback trials (Fig. 3b), affecting loss learning rates [estimated multiplier for *α*_*loss*_ = 1.19, 95% HDI = (1.0002, 1.42)] but not positive learning rates [estimated multiplier for *α*_*reward*_ = 1.004, 95% HDI = (0.90, 1.13)].

In line with results from models fit to training alone, we found weak evidence for differences in inverse temperature parameters from models fit to training-plus-test (*β*^′^), with distanced participants having an estimated 0.16 [95% HDI = (−0.05, 0.37)] and 0.18 higher *β*^′^ [95% HDI = (−0.02, 0.38)] values from single and dual learning rate models respectively. There was also evidence for differences in sensitivity to feedback from the single learning rate model additionally fit to test phase choices [estimated multiplier for *α*^′^ = 1.15, 95% HDI = (0.99, 1.33); Fig. 3a]. Results from the dual learning rate model confirmed this specificity to negative feedback, with distanced participants estimated to have 24.6% higher loss learning rates at end of training [estimated multiplier for 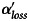 = 1.25; 95% HDI = (1.05, 1.48); Fig. 3b].

#### Temporal emergence of differences in learning parameters

**Figure 4.**
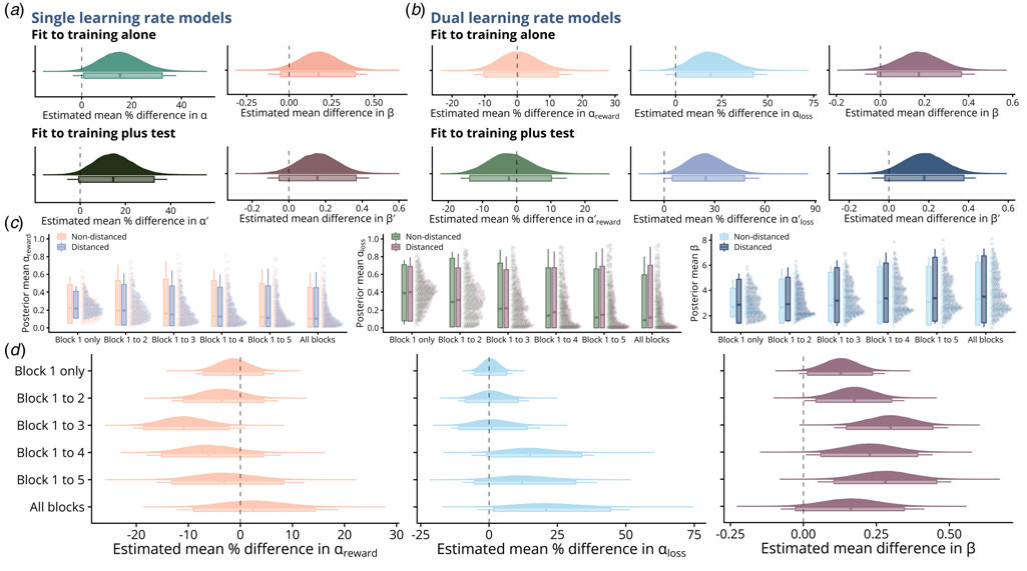
Model-derived comparisons between distanced and non-distanced participants. a–b. Coefficient posterior distributions from Bayesian GLMs (adjusted for age, gender, and digit span) reflecting the estimated percentage change in the learning rate parameter *α* (a) or *α*_*reward*_/*α*_*loss*_ (b), and the estimated mean change in the inverse temperature *β*, comparing distanced and non-distanced participants. Parameters were estimated from *Q*-learning models with single (a) or dual (b) learning rates, fit to training alone or training-plus-test (parameters from fits to training-plus-test denoted with prime). c. Individual-level posterior mean parameter estimates for *α*_*reward*_, *α*_*loss*_, and *β*, for models including increasing numbers of training blocks. d. Parameter differences between distanced and non-distanced participants, estimated from dual learning rate models fit to increasing numbers of training blocks (sixty trials per block). In all plots, boxplot boxes denote 95% HDIs, and lines denote 99% HDIs.

Although we found consistent evidence of increases in loss learning rates in distanced individuals, particularly towards the end of training, there was no evidence of a group difference in test performance on pairs that included the ‘*B*’ symbol (Kruskal–Wallis 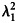 = 0.17, Holm–Bonferroni corrected *p* = 1; Fig. 2e) – the symbol with the lowest reward probability – which would be expected in the context of a higher loss learning rate (Frank et al., 2007). To investigate this, we fit dual learning rate models to increasing numbers of training blocks. As each subsequent model includes all previous trials, the results from these models cannot be interpreted directly as the best-fitting parameters for each participant over individual training blocks. However, given that learning parameters are assumed fixed in each model, estimates from models including later trials should be indicative of the underlying (dynamic) parameter values later in training. Indeed, as would be expected, reward and loss learning rate estimates decreased in both groups with the inclusion of later trials, while inverse temperatures increased (Fig. 4c).

We then compared the individual-level parameter values in the distanced and non-distanced groups with adjusted GLMs as before. We found that group differences in loss learning rates emerged as the task progressed (95% HDI for its multiplier between groups included 0 for all fits to fewer than all six blocks: Fig. 4d), with distanced participants maintaining a higher loss sensitivity relative to non-distanced participants as the task progressed (Fig. 4c). In contrast, there was strong evidence that distanced participants had consistently higher inverse temperature parameter values throughout training, with all 95% HDI excluding 0 except for the final fit to all six blocks, and some evidence that they had 10.9% lower positive learning rates over the first three training blocks [i.e. block one to three; estimated multiplier for *α*_*reward*_ = 0.89; 95% HDI = (0.81, 0.98); Fig. 4d].

## Discussion

Learning to predict rewards and avoid punishments is essential to adaptive behaviour. Disruptions in this fundamental process have been found across psychiatric disorders (Lee, 2013; Maia & Frank, 2011) and may be a target of psychological therapy (Brown et al., 2021). In a large online sample representative of the UK population in terms of age, gender, and psychiatric history, we found that a common psychotherapeutic strategy, cognitive distancing, enhanced performance in a reinforcement learning task. Our results indicate two computational mechanisms may underpin the therapeutic effects of cognitive distancing: enhanced integration, potentially coupled with subsequent exploitation of previously reinforced choices; and, with time or practice, an adaptively enhanced ability to update decisions following negative feedback.

We found that cognitive distancing was associated with heightened inverse temperatures, from the start of the task. The magnitude and evidence for group differences waned over the course of training, though we note that this is consistent with the observed increases in evidence for higher learning rates in the distancing group. Higher inverse temperatures are indicative of clearer representations of differences in true (latent) values between choice options (Pedersen & Frank, 2020), and may have enabled more deterministic choosing of the ‘better’ option in each pair, over those with less reward-certainty. Higher inverse temperature values can also be interpreted as a bias towards exploitation of current task knowledge, *v.* exploration of uncertain options (Pedersen & Frank, 2020). Notably, a reduction in inverse temperature in patients with major depressive disorder has been found in previous studies using similar tasks and computational models (Huys et al., 2013; Pike & Robinson, 2022), and we did find that higher levels of anxiety/depression symptomology were associated with slightly lower inverse temperatures, albeit with very weak evidence (Fig. 3a). Taken with evidence that cognitive distancing is an effective therapeutic strategy for depression (Kross et al., 2012), our results tentatively suggest that cognitive distancing interventions may improve depressive symptoms by increasing exploitation of rewarding outcomes. This is a key hypothesis emerging from our results, which will require confirmatory evidence from future longitudinal studies.

Distanced participants also adaptively altered reward and loss learning throughout the task, possibly adjusting to changing task dynamics. Initially, higher inverse temperatures compared to non-distanced participants suggest they more quickly developed preferences for certain symbols. However, deterministic preferences may not be the best strategy for the entire task: initial impressions can be wrong, especially for the harder-to-distinguish stimuli. Notably, towards the end of the task, distanced participants became more sensitive to negative feedback, and showed weaker evidence for inverse temperature differences, which is consistent with more exploratory behaviour later in the task. At this stage, losses may be more informative: assuming preferences are largely correct for the easier pairs, negative feedback will be rarer, and primarily experienced when choosing between the harder-to-distinguish pairs. Previous work shows that participants adjust dual learning rates independently, increasing learning rate when one type of feedback becomes more informative (Pulcu & Browning, 2017). By increasing loss sensitivity, and perhaps testing out less preferable options, distanced participants may have been able to discern values of harder-to-distinguish pairs more accurately by the end of training, enabling better performance when subsequently tested on these pairs.

A higher loss learning rate seems therapeutically counterintuitive, given several mental health disorders are marked by heightened punishment sensitivity (Elliott, Sahakian, Herrod, Robbins, & Paykel, 1997; Jappe et al., 2011; Pike & Robinson, 2022). Critically, however, aberrant loss learning may represent a ‘catastrophic response to perceived failure’ (Elliott et al., 1997; Roiser & Sahakian, 2013). In contrast, a high loss learning rate in distanced participants was accompanied by better performance. It is notable that there was limited evidence for increased learning rates in distanced relative to non-distanced participants unless later training blocks were included; indeed, there was evidence that distanced individuals had lower reward learning rates over the first half of the task. This suggests that practice may be critical to realising the beneficial effects on aspects of learning. Speculatively, our results suggest that persistent use of cognitive distancing might drive symptom change by improving patients' ability to learn from negative experiences, and apply that learning to more adaptive behaviours. Clinically, this suggests the mechanism underlying distancing may be *more effective engagement with negative information*, rather than reduced engagement with negative information.

Our finding of weak negative associations between inverse temperatures and compulsive behaviour factor scores is consistent with previous work reporting a (positive) association between compulsive behaviour and deficits in goal-directed control, characterised by the (in)ability to integrate information over time (Gillan et al., 2016). However, we only found very limited evidence for a similar negative association between inverse temperature parameters and anxiety/depression symptom scores. This result is at odds with a considerable body of literature reporting dysfunctional reinforcement learning across psychopathologies (Maia & Frank, 2011), especially depression (Halahakoon et al., 2020). That said, most previous work has compared clinical populations to healthy controls, which may result in better detection of a true effect due to greater symptomatic delineation between groups. Alternatively, together with publication bias in the field (Halahakoon et al., 2020), true effects may be smaller than those reported by small-scale clinical studies (Gelman & Carlin, 2014). Constraints on our study design and length due to its online nature may also have restricted our ability to detect associations for three reasons. Firstly, ‘loss’ trials in our task lacked actual punishment, which is experienced differently to the absence of reward (Huys et al., 2013). Secondly, we assessed symptoms through self-report questionnaires in the general population which may not be comparable to more severe clinical populations. Thirdly, we took a fully transdiagnostic approach to psychopathology (Dalgleish, Black, Johnston, & Bevan, 2020; Wise et al., 2022), which precluded us from additionally deriving categorical psychiatric measures and investigating any potential taxonic associations with learning parameters, as complete symptom questionnaires were not included for many key mental health disorders.

We note several limitations. Though motivated by discrepancies between test phase performance and preregistered computational analyses, it should be noted that the increasing-blocks analyses were exploratory, and inherently limited in that we could not estimate group differences in parameter values over individual blocks. The effects we report are generally small, perhaps reflecting the subtlety of our distancing manipulation. Note, however, that recent work finds linguistic distancing in therapy has a small but crucial clinical effect (Nook, Hull, Nock, & Somerville, 2022). These effects may also be partially mediated by expectancy (e.g. ‘placebo effects’), which some have argued play a central role in the beneficial effects of psychotherapy (Enck & Zipfel, 2019). Without consensus as to the active ingredients of psychological therapy, the potential contribution of expectancy is a limitation of any psychological therapy (Weimer, Colloca, & Enck, 2015). As noted previously, we also found only very limited evidence for often-reported negative associations between inverse temperatures and anxiety/depression psychopathology (Pike & Robinson, 2022), weakening the interpretation that distancing may act to resolve this. Lastly, we did not collect information on participants' previous experience of psychological therapies, preventing us from controlling for potential advantageous effects of prior exposure to distancing or similar psychotherapeutic techniques.

In this study, by using a relatively simple learning paradigm with an established computational model, we were able to measure the effects of cognitive distancing on well-understood choice behaviour. This echoes clinical reports that distancing causes an adaptive shift in depressed people's processing of negative experiences (Kross et al., 2012). Our work demonstrates the utility of computational approaches for reverse-translational studies to illuminate the mechanisms of existing treatments, in turn enabling improved augmentation and potentially personalisation of treatment for mental health disorders.

## Supporting information

Dercon et al. supplementary material Dercon et al. supplementary material

## Supplementary material

The supplementary material for this article can be found at https://doi.org/10.1017/S0033291723001587

## Open code and data

All data, task, and analysis code are shared openly to encourage replication and extension of our findings. Our results can be reproduced step-by-step via an R package and Jupyter notebooks – see the Github repository (https://github.com/qdercon/pstpipeline) for more details. Our study preregistration can be found on OSF (https://osf.io/fd4qu). An earlier version of this manuscript was pre-printed on PsyArXiv (https://psyarxiv.com/jmnek).
