# Supplementary material for: A core component of psychological therapy causes adaptive changes in computational learning mechanisms
Source: Psychol Med. 2023 Jun 8;54(2):327–37. doi: 10.1017/S0033291723001587 (PMC11949617; doi:10.1017/S0033291723001587)
Supplement: Dercon et al. supplementary material [file S0033291723001587sup001.pdf]

Supplementary online material

1 Supplementary methods

1.1 Representative sample

In order to sample participants who together were broadly representative of the UK in terms of age, gender, and psychiatric history, we used population data combined with Prolific[1] pre-screeners. First, the proportion of males and females in each of five age-groups (18-24, 25-34, 35-44, 45-54, 55+; chosen due to Prolific's user base skewing young in age) in the UK population was calculated based on numbers from the 2011 UK Census[2]. This was then combined with the percentage of males and females in each age group reporting any ever diagnosed psychiatric disorder taken from the 2014 Adult Psychiatric Morbidity Survey[3] to calculate targeted numbers with and without a history of any mental health condition, for males and females in each of the five age-groups, assuming a total sample size of 1,000 participants. We then used Prolific[1] pre-screeners for age, natal sex, and self-reported prior diagnosis of a psychiatric disorder, to recruit batches of the targeted numbers of UK nationals for each of the twenty groups. Overall, we were able to recruit a sample who self-reported rates of diagnosed psychiatric conditions that were similar to that of the UK population at large, with overall 6% more male and 7% fewer female participants reporting a psychiatric condition in the demographic quiz preceding the task compared with the targeted numbers (Figure S1). Note that while participants were pre-screened on Prolific for natal sex, we report and adjust in models for self-reported gender from our demographics questionnaire throughout the manuscript, though note these differed only for three participants in the sample.

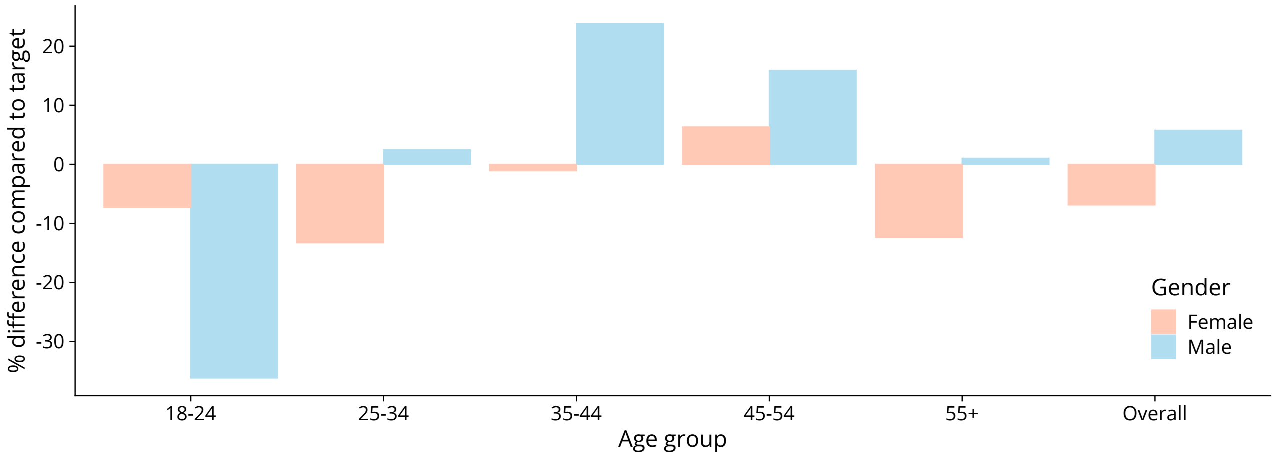

Figure S1: **Representative sampling**. Percentage difference between the numbers of males and females in each age group (excluding $n = 5$ non-binary individuals) reporting a history of any diagnosed psychiatric disorder, compared to the targeted numbers calculated from UK population data assuming 1,000 participants.

1.2 Questionnaire subsets used to predict transdiagnostic factors

As explained in the Methods, seventy-eight items from eight psychiatric questionnaires[4–11] were used to derive transdiagnostic factor scores across three symptom dimensions[12,13] (Table S1). In addition, full questionnaires were included for anhedonia (dimensional anhedonia rating scale (DARS)[14]), schizotypy (schizotypal personality questionnaire - brief revised (updated) (SPQ-BRU)[15]), body perception (body perception questionnaire - very short form (BPQ-VSF)[16]), and fatigue impact (modified fatigue impact scale (MFIS)[17]). Questionnaire order was randomised across participants, and the individual questions within each questionnaire were asked one at a time to prevent straightlining.

Table S1: Subset of 78 questionnaire questions used to predict transdiagnostic factors. For the Liebowitz social anxiety scale (LSAS), each question was asked twice to quantify both 'fear' and 'avoidance', and the average of the two taken.

Questionnaire	Question numbers (**reversed scoring**)	Scale
Obsessive compulsive inventory revised (OCIR)	2-9, 11-16, 18	0 to 4
Eeating attitudes test (EAT)	1-4, 6-8, 10-12, 14, 15, 18, 20-24	0 to 5
Apathy evaluation scale (AES)	**17**, **18**	0 to 3
Alcohol use disorders identification test (AUDIT)	3	0 to 4
Self-rating depression scale (SDS)	1, **11**, **12**, **14**, **16-18**, **20**	1 to 4
State-trait anxiety scale (STAI)	**1**, **3**, 4, 5, 8, **10**, 12, **13**, 15, **16**, 17, **19**, 20	1 to 4
Barratt impulsiveness scale (BIS)	6, **9**, **10**, **13**, 14	1 to 4
Liebowitz social anxiety scale (LSAS)	2, 6-12, 15, 16, 18-21, 23, 24	0 to 3

1.3 Digit span task

To control for working memory differences, a visual digit span task (JavaScript code adapted from this repository[18]) was administered following the probabilistic selection task probabilistic selection task (PST). Participants were instructed to memorize sequences of numbers presented one at a time (1000ms per digit). After completing a practice trial, the task began with a single digit, and sequences were extended by one digit until two sequences of the same length could not be reported, or if a sequence of twenty-five digits was correctly recalled.

1.4 Cognitive distancing instructions

The concept of cognitive distancing was introduced in a short video, which had to be watched to proceed to the task. The following written instructions provided if the participant had issues loading the video.

> "When you're doing the task, you might feel various emotions – for example, irritation, engagement, or happiness. But throughout the task, we would like you to practice a mental strategy called **self-distancing**.
>
> Self-distancing is the ability to take mental 'step back' from your immediate reactions to events, and view these events from a broader, calmer, and less emotional perspective.
>
> One way of practising this is to imagine yourself as an external observer, watching yourself perform the task from a distance, and seeing the results of each of your decisions in the task.
>
> You'll still learn which symbols win you more points than others, and you should still try to win as many points as possible. But whenever you feel irritated, happy, or any other emotion, even if it feels minor, try to distance yourself from your immediate reaction, by taking a step back from how you are feeling. We understand that this will be tricky, and so if you are unable to distance yourself from your emotional reaction on a particular trial, that is completely fine! Simply honestly report how you're feeling when we ask, and then try again to distance yourself next time."

1.5 Deviations from preregistration

The demographic exclusion criteria (low English proficiency and/or any neurological disorder), plus the catch questions were included in our preregistration, while the task-based exclusion criteria ($\geq$ 95% preference for a single key, which no one met, and digit spans of 0) were not included. Instead, we had initially opted to exclude poor performers through an accuracy criterion ($\geq$60% correct on the AB pair). However, after running the first batch of 100 participants, we found that over 35% had been excluded, largely due to the accuracy criterion. We consulted with experts in the field who had run similar studies online (including publicly via Twitter) who advised us that excluding based on accuracy may unfairly bias our sample as mental health

symptoms are commonly associated with cognitive changes[19] that could affect accuracy and suggested that we remove this exclusion criterion and replace it with the task-based exclusion criteria we used for all subsequent participants. We were also advised at this stage to add a multiple-choice quiz on the task instructions, which had to be answered correctly to begin the task, so this quiz was not completed by the first 100 participants.

1.6 Q-learning models

Models fit to training data alone

Model-free reinforcement learning in the PST is commonly modelled using Q-learning models[20–22]. In these models, the weight or Q-value $Q_t(s_t, a_t)$ for a given action a in state s at time t is an estimate of the state-action value, which can in turn be understood as an estimate of the expected sum of future rewards, conditional on that action at time t[20]. Q-values are updated trial-by-trial based on prediction errors (δ_t), where α is the learning rate. In bandit tasks such as the PST, δ_t is simply the difference between the Q-value for the chosen action (i.e., picking a certain symbol in a pair) and the observed reward r_t (1), as selecting a certain action is assumed not to affect the transition to future states[20].

$$\delta_t = r_t - Q_t(s_t, a_t) \tag{1}$$

The two established models of interest in our case were a standard Q-learning model with a single learning rate α (2), and an extended Q-learning model with dual learning rates, α_{reward} and α_{loss} (3)[21].

$$Q_{t+1}\left(s_t, a_t\right) = Q_t\left(s_t, a_t\right) + \ \alpha\delta_t \tag{2}$$

$$Q_{t+1}(s_t, a_t) = \begin{cases} Q_t(s_t, a_t) + \alpha_{reward}\delta_t & \text{if } \delta_t \geq 0, \text{ or} \\ Q_t(s_t, a_t) + \alpha_{loss}\delta_t & \text{if } \delta_t < 0 \end{cases} \tag{3}$$

In the single learning rate model, α can be interpreted as the sensitivity to recent feedback, with higher values indicating that Q-values are being rapidly updated in response to both positive and negative feedback. In the dual learning rate model, Q-values are assumed to be updated separately depending on whether δ_t is negative, which in turn occurs only when feedback is negative (i.e., $r_t = 0$). Higher α_{loss} can hence be interpreted as an increased sensitivity to recent negative feedback (and so reduced integration over trials), while higher α_{reward} values suggest increased sensitivity to recent positive feedback[21].

In both models, the Q-values can be converted to probabilities (i.e., of choosing one symbol over another) using a softmax logistic function as follows (4)

$$P_t(s_t, a_t) = \frac{1}{1 + e^{-\beta[Q_t(s_t,a_t) - Q_t(s_t,\bar{a}_t)]}}, \tag{4}$$

where $\bar{a}_t$ is the alternative (avoided) choice in the pair and β is an inverse temperature parameter, higher values of which indicate more deterministic choices. The absence of other symbols was assumed to not affect the probability of choosing one over the other on individual trials[23], so choices were assumed to follow a Bernoulli logistic distribution with the chance-of-success parameter equal to $logit[P_t(s_t, a_t)] = \beta[Q_t(s_t, a_t) - Q_t(s_t, \bar{a}_t)]$.

Models fit to training plus test data

Both the single and dual learning rate models can be extended to include test phase trials[21]. However, in the absence of feedback, Q-values are assumed to be fixed at the end of training, which means that the probability

of choosing one option over any other in the test phase is given by (5)

$$P_t^{test}(s_t, a_t) = \frac{1}{1 + e^{-\beta'[Q_{final}(s_t,a_t) - Q_{final}(s_t,\bar{a}_t)]}}, \tag{5}$$

where β' and Q_{final} are the inverse temperature parameter and Q-value at the end of training respectively.

1.7 Model validation and checks

1.7.1 Posterior predictive checks

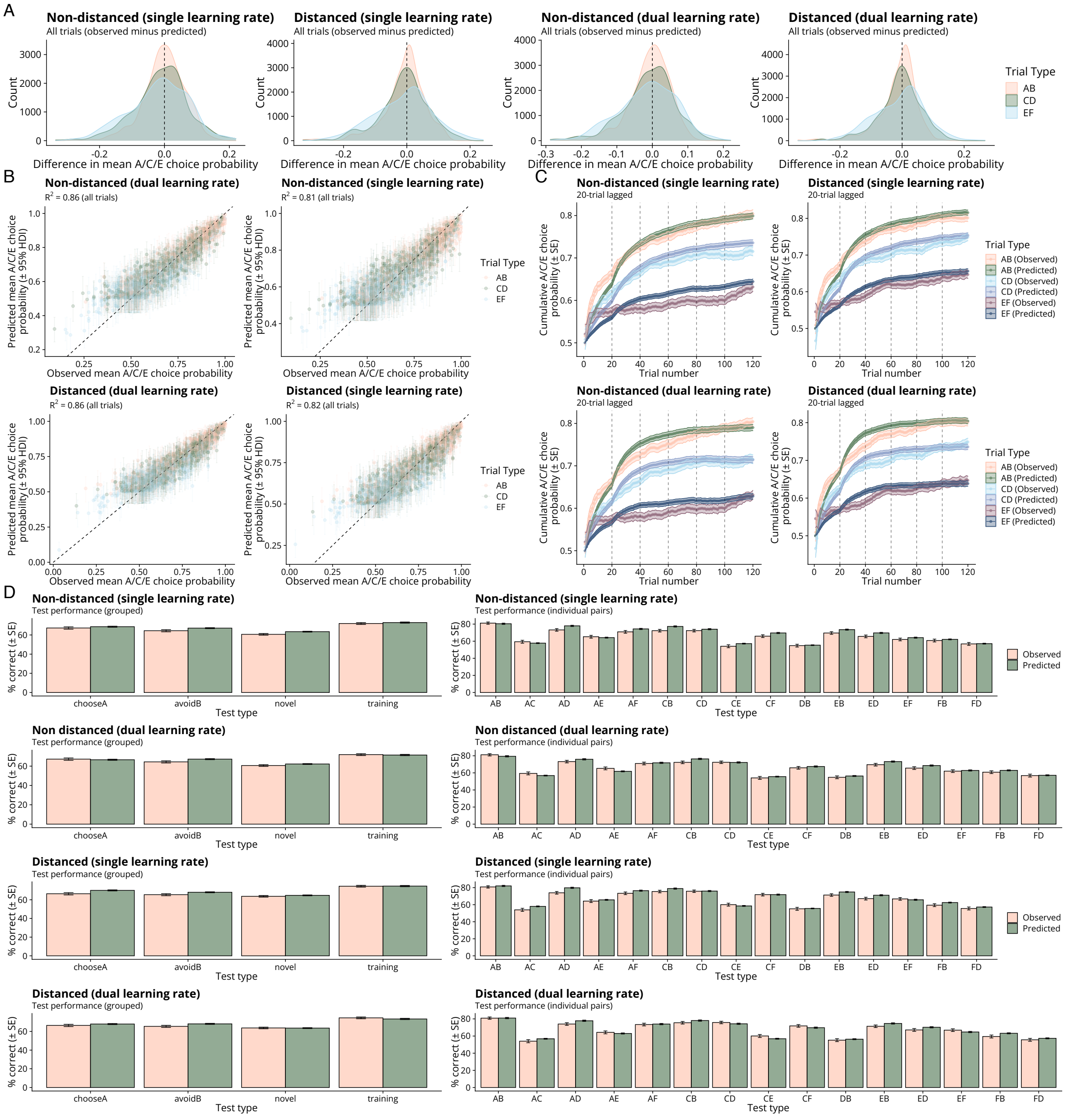

Figure S2: **Posterior predictive checks.** **A**. Difference in mean A/C/E choice probability (observed minus predicted) averaged across all trials for each individual, by symbol pair. **B**. Posterior mean A/C/E (predicted) choice probability (± 95% highest density interval (HDI)) for each individual on each pair type, plotted against the observed values after all six training blocks. **C**. Comparison between observed and predicted mean (± standard error (SE)) cumulative (twenty-trial lagged) probability of choosing the most likely correct option in each pair (symbol A, C, or E in pairs AB, CD, or EF respectively), averaged across all individuals. **D**. Predicted and observed test choices (mean % accuracy ± SE), averaged across all participants and test trials, by test trial group and for each individual test phase stimulus.

To assess the predictive validity of each model, choices were sampled from the posterior distribution for each of the 80,000 sampling iterations (20,000 draws by four chains), for each individual and trial. Choices (1 if the most likely correct symbol A/C/E was chosen for a given pair, 0 if not) were then averaged over posterior draws (i.e., $\frac{\sum_1^n choice}{n}$, where choice is 1 or 0, and n is the total number of draws) for each trial/individual to obtain the model prediction. These model-derived predictions were then visually and numerically compared to the observed data as seen below for each of the models/groups (Figure S2).

1.7.2 Parameter recovery

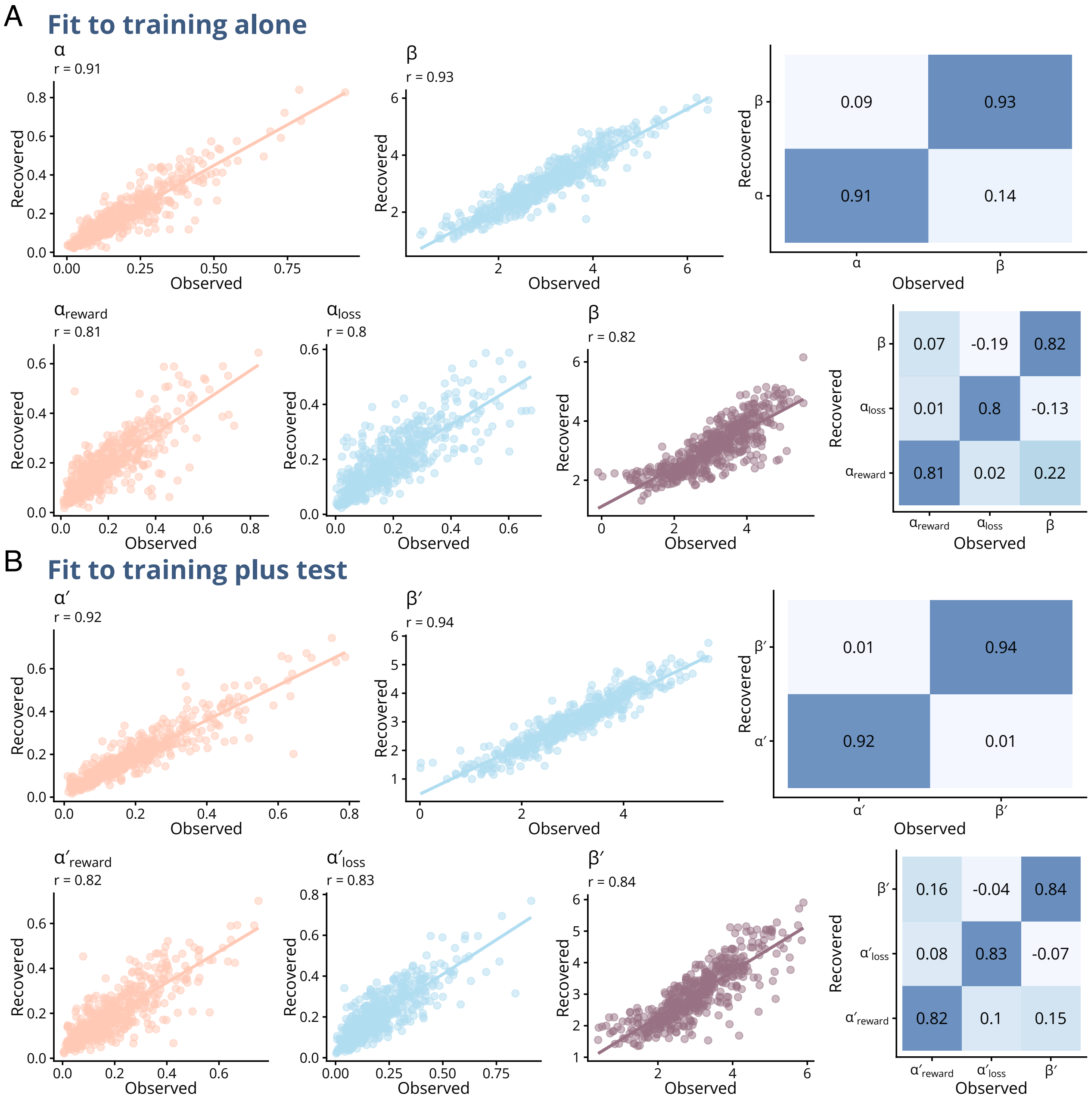

Figure S3: **Parameter recovery. A**. Plots of recovered parameter values against the observed values used to generate the task data, plus correlation plots for the single (*top*) and dual (*bottom*) learning rate models fit to training alone. **B**. Plots of recovered parameter values against the observed values used to generate the task data, plus correlation plots for the single (*top*) and dual (*bottom*) learning rate models fit to training plus test choices.

To assess whether the parameter values obtained from our models were meaningful, we simulated task data for a randomly sampled (known) set of parameter values ($n = 500$ simulated individuals per model). Learning rate parameters (α) were drawn from a $Gamma(k = 2, \Theta = 0.1)$ distribution (i.e., positively skewed,

bounded by 0), while inverse temperature parameters were drawn from a $Gaussian(\mu = 3, \sigma = 1)$ distribution. Each of the four models (i.e., single and dual learning rate models fit to training alone [Figure S3**A**], or training plus test [Figure S3**B**]) were then fit to the simulated data, and the "recovered" parameter values compared to the known parameter values for each individual.

2 Supplementary results

2.1 Multivariate associations between learning parameters and individual questionnaire items

To further investigate item-level, multivariate associations between model parameters and questionnaire items, we used Partial Least Squares (PLS) regressions[24]. PLS is a data-driven method that capitalizes on shared co-variances of multivariate data to derive underlying components and may therefore allow to identify associations not captured by the transdiagnostic symptom dimensions. In prior studies this statistical technique has been used successfully to identify transdiagnostic links between behavioural and questionnaire data[13] as well as cognitive-behavioural and neuroimaging data[25].

In line with best-practice methods to prevent over-fitting[26], we split the data into a training (75% of data) and testing set (25% of data). We first used the training data to derive the number of components that best describes the relationship between questionnaire items and model parameters in our data. This was done by comparing models with differing numbers of components on the basis of 10-fold cross-validated Mean Squared Error (MSE). The winning model was then validated out of sample in the testing data and the resulting predictive accuracy tested for significance ($\alpha = 0.05$) using permutation testing. For both the single and the dual learning rate Q-learning model, the winning model included only one component and out of sample predictive accuracy was low and non-significant (single learning rate model: MSE $= 1.550, p = 0.613$; dual learning rate model: MSE $= 0.758, p = 0.617$).

Acronyms

**AES**	Apathy Evaluation Scale
**AUDIT**	Alcohol Use Disorders Identification Test
**BIS**	Barratt Impulsiveness Scale
**BPQ-VSF**	Body Perception Questionnaire - Very Short Form
**DARS**	Dimensional Anhedonia Rating Scale
**EAT**	Eeating Attitudes Test
**HDI**	Highest Density Interval
**LSAS**	Liebowitz Social Anxiety Scale
**MFIS**	Modified Fatigue Impact Scale
**MSE**	Mean Squared Error
**PLS**	Partial Least Squares
**PST**	Probabilistic Selection Task
**OCIR**	Obsessive Compulsive Inventory Revised
**SDS**	Self-rating Depression Scale
**SE**	Standard Error
**STAI**	State-Trait Anxiety Scale
**SPQ-BRU**	Schizotypal Personality Questionnaire - Brief Revised (Updated)

References

1. Palan, S. & Schitter, C. Prolific.ac—A subject pool for online experiments. *Journal of Behavioral and Experimental Finance* **17,** 22–27 (2018).
2. Office of National Statistics. *2011 Census - Sex by age by IMD2004 by ethnic group* [Online; accessed 2021-03-17]. 2016.
3. Stansfeld, S. *et al.* in *Mental health and wellbeing in England: Adult Psychiatric Morbidity Survey 2014* 37–68 (NHS Digital, 2016).
4. Foa, E. B. *et al.* The Obsessive-Compulsive Inventory: development and validation of a short version. *Psychological Assessment* **14,** 485–496 (2002).
5. Garner, D. M. & Garfinkel, P. E. The Eating Attitudes Test: an index of the symptoms of anorexia nervosa. *Psychological Medicine* **9,** 273–279 (1979).
6. Marin, R. S., Biedrzycki, R. C. & Firinciogullari, S. Reliability and validity of the Apathy Evaluation Scale. *Psychiatry Research* **38,** 143–162 (1991).
7. Saunders, J. B., Aasland, O. G., Babor, T. F., De La Fuente, J. R. & Grant, M. Development of the Alcohol Use Disorders Identification Test (AUDIT): WHO Collaborative Project on Early Detection of Persons with Harmful Alcohol Consumption-II. *Addiction* **88,** 791–804 (1993).
8. Zung, W. W. K. A Self-Rating Depression Scale. *Archives of General Psychiatry* **12,** 63–70 (1965).
9. Spielberger, C. D., Gorsuch, R. L., Lushene, R. E., Vagg, P. R. & Jacobs, G. A. *Manual for the State-Trait Anxiety Inventory* (Consulting Psychologists Press, 1983).
10. Patton, J. H., Stanford, M. S. & Barratt, E. S. Factor structure of the Barratt Impulsiveness Scale. *Journal of Clinical Psychology* **51,** 768–774 (1995).
11. Liebowitz, M. R. Social Phobia. *Anxiety* **22,** 141–173 (1987).
12. Gillan, C. M., Kosinski, M., Whelan, R., Phelps, E. A. & Daw, N. D. Characterizing a psychiatric symptom dimension related to deficits in goal-directed control. *eLife* **5** (ed Frank, M. J.) e11305 (2016).
13. Wise, T. & Dolan, R. J. Associations between aversive learning processes and transdiagnostic psychiatric symptoms in a general population sample. *Nature Communications* **11** (2020).
14. Rizvi, S. J. *et al.* Development and validation of the Dimensional Anhedonia Rating Scale (DARS) in a community sample and individuals with major depression. *Psychiatry Research* **229,** 109–119 (2015).
15. Davidson, C. A., Hoffman, L. & Spaulding, W. D. Schizotypal personality questionnaire - brief revised (updated): An update of norms, factor structure, and item content in a large non-clinical young adult sample. *Psychiatry Research* **238,** 345–355 (2016).
16. Cabrera, A. *et al.* Assessing body awareness and autonomic reactivity: Factor structure and psychometric properties of the Body Perception Questionnaire-Short Form (BPQ-SF). *International Journal of Methods in Psychiatric Research* **27** (2018).
17. Fisk, J. D. *et al.* Measuring the functional impact of fatigue: Initial validation of the fatigue impact scale. *Clinical Infectious Diseases* **18,** S79–S83 (1994).
18. Luthra, M. & Todd, P. M. *Role of Working Memory on Strategy Use in the Probability Learning Task* in *Proceedings of the 41th Annual Meeting of the Cognitive Science Society* Montreal, Canada (2019), 721–728.
19. Millan, M. J. *et al.* Cognitive dysfunction in psychiatric disorders: characteristics, causes and the quest for improved therapy. *Nature Reviews Drug Discovery* **11,** 141–168 (2012).
20. Sutton, R. S. & Barto, A. G. *Reinforcement Learning: An Introduction* (MIT Press, 1998).
21. Frank, M. J., Moustafa, A. A., Haughey, H. M., Curran, T. & Hutchison, K. E. Genetic triple dissociation reveals multiple roles for dopamine in reinforcement learning. *Proceedings of the National Academy of Sciences of the United States of America* **104,** 16311–16316 (2007).
22. Watkins, C. J. C. H. & Dayan, P. Q-learning. *Machine Learning* **8,** 279–292 (1992).
23. Luce, R. D. *Individual Choice Behavior* (John Wiley, 1959).
24. Wold, H. Systems analysis by partial least squares (1983).
25. Kebets, V. *et al.* Somatosensory-motor dysconnectivity spans multiple transdiagnostic dimensions of psychopathology. *Biological Psychiatry* **86,** 779–791 (2019).
26. Dinga, R. *et al.* Evaluating the evidence for biotypes of depression: Methodological replication and extension of. *NeuroImage: Clinical* **22,** 101796 (2019).
